# Tackling low-value care in Singapore: progress, pitfalls, and prospects

**DOI:** 10.3389/frhs.2026.1971597

**Published:** 2026-09-30

**Authors:** Marie Ann Mae En Wong, Michael Anderson, Martin Scherer, Jose M. Valderas

**Affiliations:** 1 National University Polyclinics, Singapore, Singapore; 2 Department of Family Medicine, National University of Singapore, Singapore, Singapore; 3 Division of Population Health, Health Services Research & Primary Care, The University of Manchester, Manchester, New Hampshire, United Kingdom; 4 Department of Primary Medical Care, University Medical Center Hamburg-Eppendorf, Hamburg, Germany; 5 Department of Family and Community Medicine, University of Toronto, Toronto, Canada

**Keywords:** appropriate care, choosing wisely, healthcare waste, unnecessary care, value-based care

## Abstract

Low-value care is an important challenge for healthcare systems globally, contributing to unnecessary healthcare use and inefficient resource allocation. In Singapore, reducing low-value care is increasingly relevant given an ageing population, resource constraints and rising healthcare expenditure. However, local evidence on its prevalence, manifestations and underlying drivers remains limited. Available studies have identified potentially low-value practices in selected areas, while mechanisms established internationally, including patient expectations, clinician behaviours, financial incentives and organisational factors, may also be relevant locally but remain insufficiently characterised. Singapore has introduced initiatives to improve value and appropriateness of care, although publicly documented efforts have largely centred on public-sector and hospital-based settings, with less evidence from primary care and the private sector. Healthier SG may provide opportunities to support appropriate care through strengthened longitudinal primary care, although its impact on low-value care requires evaluation. International approaches, including evidence-based recommendations, measurement and feedback, payment mechanisms, and patient engagement provide potential lessons for local adaptation. Addressing low-value care in Singapore will require locally grounded research and coordinated, multi-component strategies across public and private sectors, accompanied by evaluation of appropriate care, outcomes, access and equity.

## 1 Introduction

Low-value care, defined as the delivery of interventions for which the added resources do not provide proportionally significant benefits (1), is a major issue for healthcare systems globally. This is particularly the case in Singapore where high levels of private sector provision, and fee-for-service incentives have contributed to concerns about overuse and inefficiency (2). While the concept of value-based care has gained prominence in Singapore (3), specific efforts to reduce low-value care in the past decade have been limited, possibly because healthcare as a percentage of GDP in Singapore remains lower than many high-income countries.

## 2 Potential drivers of low-value care in Singapore's healthcare system

Singapore is a small nation state with a high-performing healthcare system. Healthcare is widely accessible and consists of dual public-private sectors. Singapore consistently ranks well in life expectancy (84 years), infant and maternal mortality, disability-adjusted life years, and healthcare spending (4.9% of GDP in 2022) (4). However, she faces challenges such as resource limitations including finite space and manpower, and a rapidly ageing population. If healthcare expenditure grows along the current trajectory, healthcare as a percentage of GDP could increase by up to 9% by 2030 (5). Reducing low-value care is pertinent in ensuring sustainability while tackling these hurdles.

Local evidence on the prevalence of low-value care is limited, though studies have documented practices constituting low-value care. For example, an analysis of health screening packages found that 12 of 14 providers offered tests not recommended for routine screening, such as tumour markers, treadmill testing and magnetic resonance imaging (6). Recent multicentre data from public primary care clinics found low guideline concordance among antibiotic prescriptions for upper respiratory infections (7). These examples illustrate manifestations of low-value care locally, although their prevalence across the wider healthcare system has not been systematically characterised.

Mechanisms underlying low-value care in Singapore are also not well established. International literature identifies patient expectations, fear of missed diagnoses, information asymmetry, fee-for-service incentives, defensive medicine, time pressure and organisational factors (8, 9) as potential contributors to low-value care. Some of these mechanisms have also been described in Singapore healthcare research. For example, defensive medicine practices are common in Singapore due to medico-legal concerns (10), and time pressures are a frequent source of stress in clinics and hospitals (11). Organizational and system-level barriers such as lost revenue concerns (12) and inadequate decision-support infrastructure also sustain the continued provision of low-value care. There are also guideline adherence gaps and lack of standardization of care protocols between different places of practice (13). Finally, limited engagement and training in shared decision-making means patients often struggle to distinguish between necessary and unnecessary interventions (14).

## 3 Progress and pitfalls

Singapore has introduced national and institutional initiatives that aim to increase value in healthcare, although they vary in scope and extent to which reducing low-value care is an explicit objective. The national Value-Driven Care programme was instituted in 2017 and involves employing value-based methodologies to 19 high-impact conditions such as community acquired pneumonia and total knee replacements for osteoarthritis. Reported programme-level outcomes include reductions in adverse outcomes and healthcare utilization such as readmissions and reoperations (15). More recently, the Ministry of Health has positioned Appropriate and Value-Based Care as a broader system-wide priority aimed at ensuring care is clinically appropriate, evidence-based, and sustainable (16).

On the institutional level, the National University Health System has an Appropriate Care programme initiated in 2024 that aims to reduce low-value interventions through periodic educational emails sent to physicians. Singapore General Hospital also has a Value Driven Care team to analyse outcomes and costs for conditions of priority to national healthcare needs and implement best practices. Given the limited published literature describing low-value care initiatives in Singapore, the initiatives presented here are illustrative rather than an exhaustive or systematically identified set of examples.

Existing initiatives focus largely on public, hospital-based care, despite a large proportion of patients seen in primary care and the private sectors, which are areas where low-value care clearly needs to be addressed. Efforts also tend to be “top-down” where authorities implement methodologies or ideas for healthcare staff to follow. There is a lack of patient and public involvement, and behavioural interventions. While programmes such as Value-Driven Care have reported improvements in clinical and utilisation outcomes, there has been limited evaluation of interventions explicitly designed to identify or reduce low-value care as a distinct phenomenon. Empirical research characterising the prevalence, drivers and consequences of low-value care across Singapore's healthcare system remains limited.

## 4 Discussion—prospects

Healthier SG, launched by Singapore's Ministry of Health in 2023, is a long-term healthcare reform aimed at shifting the focus from hospital-based treatment to preventive and chronic care (17). Central to the initiative is the enrolment of residents with a chosen primary care provider, who develops a personalised health plan. While primarily preventive in nature, Healthier SG also offers opportunities to reduce low-value care. Its financing reforms operate at different levels: public healthcare clusters receive population-based funding, while participating GP clinics receive an annual service fee for each enrolled resident, tiered according to care needs, and preventive and chronic disease management. The latter may help align incentives towards longitudinal and preventive care rather than service volume.

Continuity of care with a regular primary care provider fosters trust, which enhances the effectiveness of right-siting and strengthens the gatekeeping role of primary care. It also reduces GP-hopping, a practice associated with duplicative and fragmented care, while facilitating patient education on the appropriateness and necessity of medical interventions. Preventive services like vaccinations and screenings are subsidised when clinically appropriate. With health plans managed electronically, low-value care metrics could be integrated into provider performance evaluations, further incentivising appropriate, high-value care. However, whether these features translate into reductions in low-value care remains to be established. Evaluation should assess reductions in low-value services alongside appropriate care, patient outcomes, access and equity, while monitoring for unintended consequences such as underuse of appropriate care, reduced access, and widening inequities.

Singapore can also draw lessons on how to reduce provision of low-value care from other high-performing healthcare systems. International examples from England, Australia, and South Korea were purposively selected, rather than identified through a systematic search, to illustrate different mechanisms for reducing low-value care operating across levels of the healthcare system, including evidence-based guidance, clinician-patient shared decision making, monitoring and accountability, and payment reform.

Choosing Wisely campaigns which are implemented in more than 20 health systems globally (18), demonstrate an approach centred on clinician-patient conversations about unnecessary tests and treatments. England's Evidence-Based Interventions (EBI) programme monitors activity for low value interventions and introduces provider and commissioner level targets to reduce provision (19). In Australia, national initiatives such as EVOLVE (20) and Better Care Everywhere (21), identify low-value practices and support clinician behaviour change across disciplines and care settings, while health services research has evaluated potentially unnecessary prescribing across different settings (22). In Asia, efforts specifically targeting low-value care remain limited (23). Broader payment reforms may nevertheless offer relevant mechanisms for addressing overuse. In South Korea, diagnosis-related group (DRG)-based payment has been implemented for selected conditions, partly to address incentives for excessive service provision under fee-for-service payment, with evaluations reporting reductions in length of stay and readmissions (24).

These approaches offer mechanisms rather than directly transferable programmes for Singapore. Their application would need to account for differences in financing, governance and delivery across public and private sectors. Table 1 translates these international lessons into potential implementation strategies for Singapore, specifying the mechanism, potential applications in Singapore Singapore, low-value care targeted, responsible actors and settings, evaluation measures, patient and public involvement, and potential unintended consequences.

**Table 1:** Potential strategies for reducing low-value care (LVC) in Singapore informed by international approaches.

Mechanism	Relevant international initiative(s)	Potential application/ intervention in Singapore	Target LVC	Responsible actor(s) & setting	Evaluation metrics	Patient/public role	Foreseeable unintended effects
Evidence-based recommendations	Choosing Wisely, EBI, EVOLVE, Better Care Everywhere	Develop locally relevant recommendations identifying tests/treatments that should be avoided or restricted to specified indications	Selected tests, treatments or procedures with evidence of inappropriate use	Professional bodies, Ministry of Health (MOH), healthcare institutions, public/private settings across primary and specialist care	LVC use, guideline-concordant appropriate use	Input into recommendation development	Underuse if recommendations applied rigidly, displacement of care to private sector
Clinician-patient shared-decision making	Choosing Wisely, EBI, Better Care Everywhere	Patient-facing resources and shared decision-making tools addressing unnecessary care	Low-value tests and treatments influenced by patient expectations or requests	Professional bodies, clinicians, public/private settings across primary and specialist care	LVC use, patient knowledge, decision quality, patient-reported experience measures	Co-design resources and participate in decisions	Perceived rationing, reduced trust, inequitable engagement
Monitoring and feedback	EBI; Better Care Everywhere	Monitor selected LVC indicators and provide feedback to providers	Measurable high-volume LVC services	MOH, healthcare institutions, providers	LVC rates, variation, outcomes	Patient-relevant outcomes included in evaluation	Gaming; target-driven behaviour, underuse
Payment reform	South Korea DRG-based payment	Expand alternative payment arrangements in settings where service-based reimbursement may incentivise unnecessary care	Low-value services potentially incentivised by volume-based reimbursement	MOH, insurers, private healthcare providers	LVC utilisation, costs, outcomes, access	Patient outcomes and experience incorporated into evaluation	Under-provision, premature discharge, patient selection, cost shifting

Given the multiple drivers of low-value care, no single intervention is likely to be sufficient. A coordinated, multi-component approach addressing both supply- and demand-side drivers, supported by sustained policy commitment and collaboration across Singapore's public and private healthcare sectors, is therefore likely to be needed (25). The approaches considered here suggest potential value in combining interventions targeting clinician and organisational practices with patient and public engagement. Evaluation of such strategies could consider their effects on low-value care alongside appropriate care, patient outcomes, access and equity, including potential unintended consequences such as underuse.

## 5 Conclusion

Singapore has established initiatives promoting value and appropriate care, but low-value care remains incompletely characterised, with limited empirical evidence on its prevalence and drivers. Existing initiatives have largely centred on public and hospital-based care, with comparatively limited attention to primary care and the private sector. Healthier SG introduces financing and continuity-of-care mechanisms that may influence low-value care, although their effects on unnecessary service use remain to be established. International experience highlights potentially relevant mechanisms, including evidence-based recommendations, shared decision-making, monitoring and feedback, and payment reform, but their applicability and effectiveness in Singapore require local evaluation. Further research is needed to establish which approaches are effective and appropriate within Singapore's healthcare context.

## Data Availability

The original contributions presented in the study are included in the article/Supplementary Material, further inquiries can be directed to the corresponding author.

## References

[1] Scott IA Duckett SJ. In search of professional consensus in defining and reducing low-value care. Med J Aust. (2015) 203(4):179–81. 10.5694/mja14.01664 26268286

[2] Matchar DB Lai WX Kumar A Ansah JP Ng YF. A causal view of the role and potential limitations of capitation in promoting whole health system performance. Int J Environ Res Public Health. (2023) 20(5):4581. 10.3390/ijerph20054581 36901591 PMC10002232

[3] Health Professionals Portal. About Appropriate & Value-Based Care (AVBC). (Accessed September 9, 2025). Available online at: https://hpp.moh.gov.sg/avbc/about/

[4] World Bank Open Data. World Bank Open Data. (Accessed 2025 September 9, 2025). Available online at: https://data.worldbank.org

[5] Singapore—Healthcare. (2025) (Accessed September 9, 2025). Available online at: https://www.trade.gov/country-commercial-guides/singapore-healthcare

[6] Yong SEF Wong ML Voo TC. Screening is not always healthy: an ethical analysis of health screening packages in Singapore. BMC Med Ethics. (2022) 23:57. 10.1186/s12910-022-00798-5 35672820 PMC9175466

[7] Koh SWC Lather S Low SH Goh JC Wong PG Cheong CKW. Diagnostic labelling, antibiotic appropriateness and watch use for upper respiratory infections in Singapore primary care: a multicentre retrospective study. J Glob Antimicrob Resist. (2026) 50:49–54. 10.1016/j.jgar.2026.05.011 42229751

[8] van Dulmen SA Naaktgeboren CA Heus P Verkerk EW Weenink J Kool RB. Barriers and facilitators to reduce low-value care: a qualitative evidence synthesis. BMJ Open. (2020) 10(10):e040025. 10.1136/bmjopen-2020-040025 33127636 PMC7604848

[9] Joshua RZ Christopher GM. Overview of the Drivers of Low-Value Care; Comment on “Key Factors that Promote Low-Value Care: Views of Experts From the United States, Canada, and the Netherlands”—Publicly Available Content Database—ProQuest. (Accessed July 28, 2026). Available online at: https://www-proquest-com.libproxy1.nus.edu.sg/publiccontent/docview/3252903951?pq-origsite = summon&sourcetype = Scholarly%20Journals

[10] Shan W. Physician sentiments on low-value investigations in Singapore: Part of Choosing Wisely campaign—Annals Singapore. (2024) (Accessed April 7, 2025). Available online at: https://annals.edu.sg/physician-sentiments-on-low-value-investigations-in-singapore-part-of-choosing-wisely-campaign/

[11] Zainal H Quah JHM Smith HE. Experiences of doctors working in Singapore’s public primary healthcare clinics. Health Soc Care Community. (2022) 30(5):e1948–58. 10.1111/hsc.13626 34725885

[12] Tan HY Tan JK Lam LT Tan DH Chua YX. Insights into Singapore’s national strategy for primary care reform: physician attitudes, facilitators and barriers to healthier SG. BMC Health Serv Res. (2025) 25:1021. 10.1186/s12913-025-13143-4 40754525 PMC12320328

[13] Tan KBH. Assessing doctors’ compliance with guidelines on diabetes management. Int J Health Care Qual Assur Inc Leadersh Health Serv. (2006) 19(2–3):267–86. 10.1108/09526860610661482 16875107

[14] Guo H Lye DC Ng TM Somani J Kwa ALH Chung SJ. Hospitalised patients as stewards of their own antibiotic therapy: a qualitative analysis informing the strategic design of interventions to encourage shared decision-making in tertiary hospital settings in Singapore. Front Public Health. (2024) 12:1347764. 10.3389/fpubh.2024.1347764 39145162 PMC11322571

[15] Ministry of Health. SPEECH BY MR ONG YE KUNG, MINISTER FOR HEALTH AND COORDINATING MINISTER FOR SOCIAL POLICIES, AT THE IHI-BMJ INTERNATIONAL FORUM ON QUALITY AND SAFETY. (Accessed September 9, 2025). Available online at: https://www.moh.gov.sg/newsroom/speech-by-mr-ong-ye-kung–minister-for-health-and-coordinating-minister-for-social-policies–at-the-ihi-bmj-international-forum-on-quality-and-safety-/

[16] Ministry of Health, Singapore. A PRIMER ON APPROPRIATE AND VALUE-BASED CARE (AVBC) IN SINGAPORE.

[17] Ministry of Health. Healthy living. (Accessed September 16, 2025). Available online at: https://www.moh.gov.sg/staying-healthy/healthy-living/

[18] Born KB Levinson W. Choosing wisely campaigns globally: a shared approach to tackling the problem of overuse in healthcare. J Gen Fam Med. (2018) 20(1):9–12. 10.1002/jgf2 30631653 PMC6321837

[19] Anderson M Molloy A Maynou L Kyriopoulos I McGuire A Mossialos E. Evaluation of the NHS England evidence-based interventions programme: a difference-in-difference analysis. BMJ Qual Saf. (2023) 32(2):90–9. 10.1136/bmjqs-2021-014478 35393354 PMC9887378

[20] Physicians TRAC of. The Royal Australasian College of Physicians. The Royal Australasian College of Physicians; (Accessed September 9, 2025). Available online at: https://www.racp.edu.au/evolve/home

[21] Better Care Everywhere initiative | Australian Commission on Safety and Quality in Health Care. (Accessed September 9, 2025). Available online at: https://www.safetyandquality.gov.au/our-work/better-care-everywhere

[22] Riess S Lei S Huang L O’Loughlin R Hiscock H. Non-indicated acid-suppression prescribing in a tertiary paediatric hospital: an audit and costing study. J Paediatrics Child Health. (2019) 55(7):762–71. 10.1111/jpc.14287 30484922

[23] Yeo JX Tokuda Y Sasaki S Pan J Kamaruzaman H Ranganathan P. Addressing low-value care (LVC) in Asia: a narrative review of choosing wisely and other initiatives across Asia. BMJ Open Qual. (2026) 15(2):e003806. 10.1136/bmjoq-2025-003806 42082281 PMC13141114

[24] Choi JW Kim SJ Park HK Jang SI Kim TH Park EC. Effects of a mandatory DRG payment system in South Korea: analysis of multi-year nationwide hospital claims data. BMC Health Serv Res. (2019) 19(1):776. 10.1186/s12913-019-4650-8 31666066 PMC6822472

[25] Colla CH Mainor AJ Hargreaves C Sequist T Morden N. Interventions aimed at reducing use of low-value health services: a systematic review. Med Care Res Rev. (2017) 74(5):507–50. 10.1177/1077558716656970 . 27402662

